# Effects of Preoperative Oral Carbohydrate on Cirrhotic Patients under Endoscopic Therapy with Anesthesia: A Randomized Controlled Trial

**DOI:** 10.1155/2021/1405271

**Published:** 2021-09-08

**Authors:** Yan Wang, Yichun Tu, Zhenglv Liu, Hui Li, Hongtan Chen, Baoli Cheng, Xiangming Fang

**Affiliations:** ^1^Department of Anesthesiology, The First Affiliated Hospital, College of Medicine, Zhejiang University, Hangzhou, China; ^2^Department of Anesthesiology, Huili Li Eastern Hospital, Ningbo, China; ^3^Department of Digestive Medicine, The First Affiliated Hospital, College of Medicine, Zhejiang University, Hangzhou, China

## Abstract

Preoperative fasting causes significant perioperative discomfort in patients. Preoperative oral carbohydrate (POC) is an important element of the enhanced recovery after surgery protocol, but its effect on cirrhotic patients who tend to have abnormal gastric emptying remains unclarified. We investigated the influence of POC on gastric emptying and preprocedural well-being in cirrhotic patients. A prospective, randomized, controlled study of cirrhotic patients with gastroesophageal varices scheduled for elective therapeutic endoscopy under intravenous anesthesia was conducted. We enrolled 180 patients and divided them into three groups: those not supplemented with carbohydrates for 8 h before therapeutic endoscopy (control group) and those administered a carbohydrate beverage 2 h (2 h group) and 4 h (4 h group) before endoscopy. The residual gastric volume was quantified before anesthesia, gastric emptying was evaluated using gastric ultrasonography, and preprocedural well-being was assessed using the visual analogue scale (VAS). Preanesthesia gastric sonography scores were similar among the three groups. No patient had residual gastric volume > 1.5 ml/kg in the control and 4 h groups, but six patients (11%) had a residual gastric volume of >1.5 ml/kg in the 2 h group, hence were at a risk of regurgitation and aspiration. Moreover, VAS scores for six parameters (thirst, hunger, mouth dryness, nausea, vomiting, and fatigue) in the 2 h group and three parameters (thirst, hunger, and mouth dryness) in the 4 h group were significantly lower than those in the control group, suggesting a beneficial effect on cirrhotic patients' well-being. Preoperative gastric peristaltic and operation scores, postoperative complications, length of hospital stay, and in-hospital expenses were not significantly different among the three groups. Our study indicated that avoiding preoperative fasting with oral carbohydrates administered 4 h before anesthesia can be achieved in cirrhotic patients. Further studies to assess whether POC can help improve postoperative outcomes in cirrhotic patients are needed.

## 1. Introduction

Liver cirrhosis is the end stage of chronic liver disease, and it is characterized by the accumulation of the fibrotic tissue and abnormal regenerative nodules [1, 2]. Variceal hemorrhage is the most life-threatening complication of liver cirrhosis and is associated with increased mortality, particularly in patients with hepatic decompensation [3, 4]. Therapeutic endoscopy is the recommended standard of care for the treatment and prevention of gastroesophageal variceal bleeding. However, it requires preprocedural fasting [5, 6]. Preoperative oral carbohydrates (POC), which avoid preoperative fasting, are widely adopted as part of the enhanced recovery after surgery (ERAS) protocol, which has shown beneficial effects in improving perioperative well-being [7]. Studies have also revealed that POC can reduce nitrogen losses, attenuate the magnitude of postoperative insulin resistance, and improve muscular strength, which result in better clinical outcomes [8].

However, patients with liver cirrhosis often show decreased gastric motility and prolonged gastric emptying, which are closely related to abnormalities in the autonomic function and portal hemodynamics [9]. There is a lack of evidence regarding the effect of POC on some individual constituents with a propensity for delayed gastric emptying, particularly in patients with cirrhosis [10]. In addition, for patients with cirrhosis, given the rapid development of the catabolic state caused by starvation, avoiding long-term fasting is an essential element of perioperative management [11]. Therefore, there is an urgent need to determine a specific duration of fasting before anesthesia in such patients [12, 13].

We designed a randomized controlled trial (RCT) to assess the effect of POC, compared with preoperative fasting, on gastric emptying, preoperative well-being, hemodynamic changes, and clinical outcomes in patients with cirrhosis subjected to therapeutic endoscopy under anesthesia. We hypothesized that avoiding preoperative fasting with oral carbohydrates administered 4 h prior to anesthesia can be achieved in patients with cirrhosis.

## 2. Methods

### 2.1. Study Design and Subjects

This trial was approved by the Ethics Committee of The First Affiliated Hospital, College of Medicine, Zhejiang University (IIT2018-940, September 10, 2018) and registered at Clinicaltrials.gov under the number ChiCTR1800018328 (September 11, 2018). The study protocol was performed in accordance with the relevant guidelines. This study was designed as a prospective RCT. The trial was conducted at The First Affiliated Hospital, College of Medicine, Zhejiang University, China. A total of 196 adult patients with cirrhosis and gastroesophageal varices hospitalized for elective therapeutic endoscopy under anesthesia between February 2019 and September 2019 were assessed for eligibility. Patients with cirrhosis were defined as those who had widespread disruption of the normal liver structure by fibrosis and the formation of regenerative nodules caused by various chronic progressive conditions affecting the liver. This study was limited to participants with American Society of Anesthesiologists (ASA) grades III to IV. We excluded patients who were at the acute variceal hemorrhage stage and those who had severe anemia (hemoglobin less than 70 g/L), a known or predicted difficult airway, moderate or severe heart and lung function impairment, asthma, or hepatic encephalopathy. A total of 180 patients were selected for the randomization, and informed consent for participation was obtained from each patient or their immediate relatives.

### 2.2. Sample Size Calculation

PASS statistical software was used to calculate the sample size. Based on our pilot study, the means and standard deviations (SD) of the visual analog scale (VAS) scores for thirst were 2.75 ± 1.15 (control group), 1.45 ± 1.22 (2 h group), and 1.9 ± 1.51 (4 h group). Therefore, a minimal sample size of 63 participants (21 per group) was required, with a two-tailed *α* = 0.05 and a power of 90% to guarantee such results. Dropout patients included those who had no time to commit to the study, did not want to drink carbohydrates, or could not complete the consumption of oral carbohydrates. With the assumption that 10% of the participants were to drop out, a minimum sample size of 69 participants was established.

### 2.3. Intervention

The enrolled patients were randomly assigned to one of the three groups: the control and 2 h and 4 h groups (*n* = 60). The randomization schedule was generated using Stata statistical software by a statistician. Randomization was stratified by site using randomly permuted blocks (block size 7), and the sizes of the blocks and the allocation sequence were known only to the data coordinating center. The control group received nothing for 8 h prior to gastroscopy. The intervention group included participants who were administered oral rehydration solutions containing carbohydrates and sodium (355 ml) 2 h or 4 h prior to the scheduled endoscopic procedure. On the day of the operation, the anesthesiologists were blinded after assignment to interventions. After receiving the interventions, 63 patients were excluded due to long operation time (>2 h), canceled operation, being sent to the intensive care unit (ICU), or conversion to open surgery. For patients with uncontrolled bleeding or punching during endoscopy interventions, there was a conversion to open surgery. Patients with unstable hemodynamic changes or those who underwent tracheal intubation due to low oxygenation during endoscopy were admitted to the ICU. In control group, 5 were excluded due to long operation time, 9 due to cancelled operation, 6 due to transfer to the ICU, and 1 due to conversion to open surgery. In the 2 h group, 2 were excluded due to long operation time, 1 due to cancelled operation, 1 due to transfer to the ICU, and 2 due to conversion to open surgery. In the 4 h group, 6 were excluded due to long operation time, 9 due to cancelled operation, 8 due to transfer to the ICU, and 13 due to conversion to open surgery (Supplemental file 1).

On arrival in the operating room, the patients were regularly monitored for heart rate (HR), blood pressure, and SpO_2_. All patients were administered oxygen at 3 L/min through a nasal cannula during the endoscopy procedure. With the patients in the left lateral position, intravenous propofol (1.5-2 mg/kg) and sufentanil (0.1 ug/kg) were administered. A stable depth of anesthesia in which patients were unconscious and unresponsive to painful stimulation was targeted by maintaining the anesthesia with propofol at a rate of 3-9 mg·kg^−1^·h^−1^ during the endoscopy. Blood pressure was recorded intermittently every 5 min, with continuous recording of SpO_2_ and HR. Atropine (0.2-0.5 mg) was administered when the HR was <50/min. Ephedrine or phenylephrine was injected when the blood pressure was <90/60 mmHg or decreased by 30% from the baseline value. If the blood pressure was >160/100 mmHg and the effects of anesthesia depth and surgical complications were excluded, urapidil was administered.

### 2.4. Measurement Outcomes

The primary outcome measures were gastric emptying assessed by gastric sonography, the volume of the residual gastric contents aspirated by gastroscopy, and the general well-being of the patients as assessed by the VAS scores for six parameters (thirst, hunger, mouth dryness, nausea, vomiting, and weakness). Secondary outcomes included hemodynamic changes, gastric peristalsis, postoperative complications, and length of hospital stay (LOS).

Gastric ultrasound is a reliable diagnostic tool for assessing the gastric contents and their volumes [14]. A standardized gastric scanning protocol was applied prior to anesthesia. We proposed a 3-point grading system based on the qualitative sonographic assessment of the antrum in the supine and right lateral positions, which correlated well with the predicted gastric volume. Patients were classified as follows: grade 0, empty antrum in both the supine and right lateral positions, corresponding to a completely empty stomach; grade 1, minimal fluid volume detected only in the right lateral position, suggesting a negligible fluid volume, mostly less than 100 ml; and grade 2, antrum clearly distended with fluid visible in both supine and right lateral positions, correlating with significantly higher fluid volumes (>100 ml) and a higher risk of regurgitation of gastric contents on induction of anesthesia [15]. Patients whom were evaluated as grade 2 underwent gastric endoscopy to collect the residual volume through a collection bottle before anesthesia for safety. Lidocaine gel and midazolam 0.03 mg/kg were administered prior to gastroscopy to ensure the adaption of patient to the procedure and to decrease anxiety and discomfort. Immediately after collecting the stomach content, anesthesia with propofol was induced. For all patients, we calculated the exact volume of the fluid, which is compatible with the risk of gastric aspiration. We assessed the relation of gastric volume to weight, and a value > 1.5 ml/kg was considered a risk factor for bronchoaspiration [16].

Patients' general well-being (thirst, hunger, mouth dryness, nausea, vomiting, and fatigue) was measured using the VAS. VAS scores of 0–10 were assessed before administering intravenous anesthesia [17]. A score of 0 indicated that the patient had no discomfort at all, while a score of 10 indicated that the patient had the most severe discomfort. All VAS scores were determined by a blinded investigator.

Hemodynamic stability was assessed by measuring the mean arterial pressure (MAP), SpO_2_, and HR at the following time intervals: immediately before intravenous anesthesia (T0), 1 min after the injection of propofol (T1), and 5 mins after the injection of propofol (T2).

Gastric peristalsis was evaluated using a 4-grade scale as such grade 1, no peristalsis; grade 2, mild peristalsis in which a peristaltic wave is formed without reaching the pyloric ring; grade 3, moderate peristalsis in which a pronounced peristaltic wave is formed and reaches the pyloric ring; and grade 4, vigorous peristalsis with a deep peristaltic wave that strangulates the antrum [18]. The operation was also scored on a 4-grade scale by an endoscopic operator based on our previous study. When operation score was more than 2, administration of an intravenous antispasmodic agents was required.

Follow-up of patients was performed on the 1^st^, 2^nd^, and 5^th^ days after the endoscopic therapy. Postoperative complications (fever and bleeding), LOS, and in-hospital expense were recorded.

### 2.5. Statistical Analysis

In our study, primary outcomes included gastric emptying assessment and VAS score (thirst) evaluation. SPSS (version 20.0) statistical software was used to analyze the data. Baseline variables were summarized with descriptive statistics. Continuous variables are presented as mean ± SD or median and interquartile ranges (IQR; 25^th^-75^th^ percentiles), as appropriate. Categorical variables are shown as numbers (*n*) and percentages (%). Between-group differences were evaluated using the *X*^2^ test or analysis of variance (ANOVA) for continuous variables. The chi-squared test or Fisher's exact test was used for the comparison of categorical variables. The variables in each group were compared using ANOVA with Bonferroni's correction. Differences were considered significant at *p* < 0.017 in ANOVA with Bonferroni's correction and at *p* < 0.05 in other analyses.

## 3. Results

### 3.1. Basic Characteristics of Patients

As shown in Table 1, the age, sex, BMI, ASA classification, duration of the endoscopic therapy, and the number of patients receiving ephedrine or phenylephrine were not significantly different among the three groups.

### 3.2. Evaluation of the Residual Fluid in the Stomach before Anesthesia

Table 2 shows a summary of the evaluation of the residual fluid in the stomach by gastric ultrasound grade and gastric content volume aspirated by gastroscopy before anesthesia. In the control group, 37 patients (94.9%) were grade 0, and two were grade 1. In the 2 h group, 48 patients (88.9%) were grade 0, five were grade 1, and one was grade 2. In the 4 h group, 22 patients (91.7%) were grade 0, and two were grade 1. There were no significant differences among the groups with regard to the gastric ultrasound score. No patient had residual gastric volume > 1.5 ml/kg in the control and 4 h groups, but six patients (11%) had a residual volume of >1.5 ml/kg in the 2 h group. No patient had any episode of regurgitation of gastric content during the course of anesthesia.

### 3.3. VAS Scores Assessed before Anesthesia

Subjective feelings of discomfort were measured during the preoperative period for six parameters using VAS scores (thirst, hunger, mouth dryness, nausea, vomiting, and fatigue). The 2 h group experienced significantly less thirst, hunger, mouth dryness, nausea, vomiting, and fatigue compared to the control group. In addition, VAS scores for thirst, hunger, and mouth dryness were significantly lower in the 4 h group than in the control group, whereas no difference was found for nausea, vomiting, and fatigue (Table 3).

### 3.4. Changes in Hemodynamics

**Figure 1:**
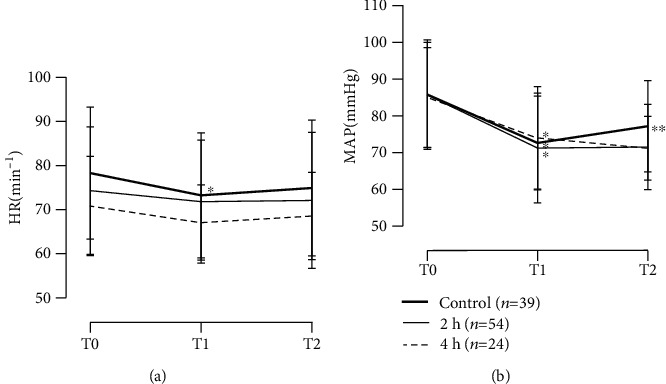
Changes in (a) heart rate and (b) mean arterial pressure. Data are expressed as means ± SD. Bold solid line, control group (*n* = 39); thin solid line, 2 h group (*n* = 54); and broken line, 4 h group (*n* = 24). ^∗^*p* < 0.001 compared with level at T0; ^∗∗^*p* < 0.05 compared with level at T1; T0, just before intravenous anesthesia; T1, 1 minute after injection of propofol; and T2, 5 minutes after injection of propofol; HR: heart rate; MAP: mean arterial pressure.

We collected hemodynamic measurements at three time points. SpO_2_ remained unchanged throughout the observation period in all the three groups (Table 4). The HR was significantly lower at T1 than at T0 (*p* < 0.001), but did not change significantly between T1 and T2 in the control group (Figure 1(a)). The HR remained unchanged throughout the observation period in the 2 h and 4 h groups. As shown in Figure 1(b), MAP decreased significantly at T1 compared to T0 in all three groups (*p* < 0.001). Moreover, MAP changed significantly between T1 and T2 in the control group (*p* < 0.05), but there was no significant difference between T1 and T2 in the 2 h and 4 h groups.

### 3.5. Gastric Peristaltic Score and Operation Score Assessment before Therapeutic Endoscopy

We calculated the gastric peristaltic score and found that there were no significant differences among the groups. In addition, the operation scores of the subjects in the 2 h and 4 h groups were similar to those of the subjects in the control group (Table 5).

### 3.6. Convalescence

A summary of the postoperative rehabilitation is shown in Table 6. In total, nine patients experienced postoperative adverse events. Bleeding complications after therapeutic endoscopy were observed in four patients: two patients in the control group and two others in the 4 h group. Fever after therapeutic endoscopy was observed in three patients in the control group, one patient in the 2 h group, and one patient in the 4 h group. Postoperative bleeding and fever rates did not differ significantly among the three groups. The LOS was 9.15 ± 4.37 days for the control group, 8.26 ± 4.34 days for the 2 h group, and 8.13 ± 5.24 days for the 4 h group. No significant difference was detected in the LOS among the three groups. Hospitalization expenses were 29147 ± 19365 RMB in the control group, 31810 ± 30962 RMB in the 2 h group, and 20643 ± 9585 RMB in the 4 h group. Hospitalization expenses were similar among the three groups.

## 4. Discussion

The current study showed that preoperative carbohydrates administered 4 h prior to anesthesia could improve the well-being of patients with cirrhosis without increasing the risk of gastroesophageal reflux and aspiration pneumonia, thus suggesting the safety and promising role of POC in patients with cirrhosis. This study also suggests that the intake of POC has beneficial effects on hemodynamic stability. Other aspects studied showed no significant differences regardless of gastric peristalsis or postoperative complications.

Patients with liver cirrhosis often show gastric dysmotility associated with prolonged gastric emptying and decreased gastric wall compliance. Delayed gastric emptying may cause disturbances in postprandial glucose, insulin, and ghrelin levels and further results in low energy intake, contributing to malnutrition and increased morbidity [19–21]. To date, there are no data to support a direct relationship between the duration of fasting and the risk of pulmonary aspiration in patients with cirrhosis. Additionally, there is a lack of universal practice standard for preprocedural fasting in patients with cirrhosis. Therefore, understanding the evidence-based preoperative carbohydrate recommendations that might impact the well-being and clinical outcomes of cirrhotic patients is of utmost importance. For the first time, we conducted a RCT to determine the timing of oral intake before anesthesia in patients with cirrhosis. When gastric emptying is impaired, such as in patients with cirrhosis, the potential for pulmonary aspiration of gastric contents must be considered when determining a specific time period of fasting before anesthesia [13]. Therefore, in this study, to confirm the safety of POC in patients with cirrhosis, endoscopic examiners performed gastroscopy and aspirated the stomach contents before anesthesia. We then measured and analyzed the volume of the gastric contents as a primary outcome parameter, which is an important factor in estimating the severity of aspiration and regurgitation. Interestingly, we found that no patient had residual fluid > 1.5 ml/kg in the control and 4 h groups. However, notably, six patients (11%) had a residual volume of >1.5 ml/kg in the 2 h group, indicating a high risk of regurgitation. Based on these findings, we suggest that preoperative carbohydrates administered 4 h rather than 2 h prior to anesthesia may be safer in patients with cirrhosis. Our study adds to the knowledge on preoperative fasting guidelines for anesthesia in patients with cirrhosis.

In this study, we further examined the effect of preoperative carbohydrate supplementation on the stresses caused by endoscopic examination associated with fasting in patients with cirrhosis. Importantly, we noted lower preoperative VAS scores for thirst, hunger, mouth dryness, nausea, vomiting, and fatigue in the 2 h carbohydrate group than in the control group. In addition, the VAS scores for thirst, hunger, and mouth dryness were significantly lower in the 4 h group than in the control group. These findings suggest that preoperative carbohydrate loading is acceptable. This is in agreement with previous reports which found that patients had an effectively reduced thirst, hunger, nausea, and vomiting, as main components of preoperative discomfort, when consuming carbohydrates before surgery [22, 23]. Our findings are also consistent with those of a study that demonstrated that 200 kcal supplementation could reduce both self-assessed physical and mental stresses that are associated with fasting in patients with cirrhosis who are required to undergo contrast-enhanced CT or contrast-enhanced MRI [24]. Furthermore, to the best of our knowledge, malnutrition is common in patients with cirrhosis, and its reported prevalence is as high as 80%. Low energy intake and poor nutritional status facilitate the development of hepatic encephalopathy, which is associated with poor prognosis in patients with cirrhosis [24]. Therefore, avoiding long-term fasting with the intake of carbohydrates 4 h prior to anesthesia may have beneficial effects on ameliorating malnutrition in patients with cirrhosis; however, further research is warranted [25, 26].

To date, esophageal and gastric variceal bleeding is considered the major cause of upper gastrointestinal hemorrhage in patients with cirrhosis, with a high risk of mortality and poor prognosis. It is therefore essential that patients with liver cirrhosis should not only receive interventions to survive acute variceal hemorrhage but also undergo secondary prophylaxis [27]. Advancements in multidisciplinary approaches, including pharmacological therapy, endoscopic intervention, transjugular intrahepatic portosystemic shunt, and surgery, have improved the outcomes in patients with cirrhosis. Therapeutic endoscopy has great clinical value in achieving hemostasis and preventing first as well as recurrent bleeding from esophageal and isolated gastric varices in patients with cirrhosis [28–30]. Although therapeutic endoscopy is a relatively quick procedure, the choice of sedation and anesthesia selected for patients with cirrhosis continues to be a controversial issue. In our study, we used propofol in combination with opioids to keep patients under moderate or deep sedation during the therapeutic endoscopy. Among 117 patients, no patient showed gastroesophageal reflux or aspiration pneumonia that was caused, indicating that propofol-based sedation with appropriate monitoring could be safe during therapeutic endoscopy in patients with cirrhosis. This is in agreement with previous reports that found that the use of propofol for sedation was safe during colonoscopy for patients with liver cirrhosis [31].

This study had some limitations. First, the long-term effect of preoperative carbohydrate intake in patients with liver cirrhosis is unclear. Second, large multicenter RCTs will be needed to further conform propofol-based sedation is the best choice for this subgroup of patients. Third, only data of routine clinical variables were used in the present study, and we expect that specific assessments, including nutritional and metabolic status, can be incorporated into future studies. Fourth, we did not classify patients with cirrhosis with different Child-Pugh scores into different subgroups, and further research is needed to verify the specific preoperative fasting time among the various subgroups of patients with cirrhosis. Fifth, the exclusion rate of each group was not quite low, indicating larger amounts of patients should be involved in further research.

## 5. Conclusion

In summary, we found that avoiding preoperative fasting by consuming carbohydrates 4 h prior to anesthesia has considerable advantages in improving the well-being of patients with cirrhosis undergoing therapeutic endoscopy, without increasing their risk of regurgitation. Our findings suggest more favorable outcomes if carbohydrates are taken 4 h preoperatively in patients with cirrhosis. Our data add new insights to be considered in future evidence-based guidelines for preoperative fasting in anesthesia for patients with cirrhosis.

## Figures and Tables

**Table 1:** Basic characteristics of patients.

	Control	2 h	4 h	*p*
Age (year, mean ± SD)	54.87 ± 10.25	55.85 ± 9.43	58.54 ± 11.09	0.366
Female (*n*, %)	11 (28.2)	17 (31.5)	4 (16.7)	0.395
BMI (kg/m^2^, mean ± SD)	22.97 ± 3.08	22.40 ± 2.53	22.68 ± 1.92	0.593
ASA (III/IV, *n*)	7/32	10/44	2/22	0.546
Duration of surgery (min, mean ± SD)	27.15 ± 13.98	34.57 ± 21.65	34.96 ± 15.71	0.113
Receiving ephedrine (yes, %)	16 (41)	18 (33.3)	7 (29.2)	0.593
Receiving phenylephrine (yes, %)	1 (2.6)	7 (13.0)	1 (4.2)	0.150

Note: BMI: body mass index; ASA: American Society of Anesthesiology.

**Table 2:** Evaluation of residual fluid in the stomach before anesthesia.

	Control	2 h	4 h	*p*
Gastric sonography score (*n*, %)				0.586
0	37 (94.9)	48 (88.9)	22 (91.7)	
1	2 (5.1)	5 (9.3)	2 (8.3)	
2	0 (0)	1 (1.8)	0 (0)	
Fluid sucked during gastroscopy (*n*, %)				0.043
<1.5 ml/kg	39 (100)	48 (88.9)	24 (100)	
a ≥1.5 ml/kg	0 (0)	6 (11.1)	0 (0)	

Note: the variables in each group were compared using ANOVA with Bonferroni's correction.

**Table 3:** Well-being by VAS score before anesthesia.

	Control	2 h	4 h	*p*
Thirst (mean ± SD)	3.69 ± 1.58	1.83 ± 1.20	2.94 ± 1.32	<0.05^a,b^
Hunger (mean ± SD)	4.21 ± 1.74	2.94 ± 1.50	2.54 ± 1.67	<0.05^a,b^
Mouth dryness (mean ± SD)	4.41 ± 1.77	3.44 ± 1.76	2.50 ± 1.32	<0.05^a,b^
Nausea (mean ± SD)	1.13 ± 2.18	0.09 ± 0.68	0.75 ± 0.99	<0.05^a^
Vomiting (mean ± SD)	1.13 ± 2.18	0.09 ± 0.68	0.75 ± 0.99	<0.05^a^
Fatigue (mean ± SD)	4.26 ± 2.00	2.42 ± 0.97	3.52 ± 1.55	<0.05^a^

Note: the variables in each group were compared using ANOVA with Bonferroni's correction. *p* < 0.05^a^: control group vs. 2 h group; *p* < 0.05^b^: control group vs. 4 h group.

**Table 4:** SpO2 changes at three time points.

	Control	2 h	4 h	*p*
T0^∗^	99 (98, 100)	100 (99, 100)	99 (98, 100)	0.194
T1^∗∗^	100 (99, 100)	100 (98, 100)	99 (98, 100)	0.302
T2^∗∗∗^	100 (99, 100)	100 (99, 100)	100 (98, 100)	0.479

Note: ^∗^just before intravenous anesthesia; ^∗∗^1 minute after injection of propofol; ^∗∗∗^5 minutes after injection of propofol.

**Table 5:** Evaluation of gastric peristaltic score and operation score.

	Control	2 h	4 h	*p*
Gastric peristaltic score	1.85 ± 0.54	1.65 ± 0.48	1.87 ± 0.68	0.172
Operation score	1.90 ± 0.60	1.65 ± 0.48	1.87 ± 0.68	0.114

**Table 6:** Postoperative complication and LOS.

	Control	2 h	4 h	*p*
Postoperative bleeding rate (no/yes)	37/2	54/0	22/2	0.066
Postoperative fever rate (no/yes)	36/3	53/1	23/1	0.356
LOS time (day)	9.15 ± 4.37	8.26 ± 4.34	8.13 ± 5.24	0.319
Hospitalization expenses (RMB)	29147 ± 19365	31810 ± 30962	20643 ± 9585	0.220

Note: LOS: length of hospital stay; RMB: Ren Min Bi.

## Data Availability

The datasets used during the current study are available from the corresponding author on reasonable request.

## Supplementary Materials

Supplementary Materials Supplemental file 1 shows flow diagram of this randomized controlled trial.
