# Supplementary material for: Effects of Preoperative Oral Carbohydrate on Cirrhotic Patients under Endoscopic Therapy with Anesthesia: A Randomized Controlled Trial
Source: Biomed Res Int. 2021 Sep 8;2021:1405271. doi: 10.1155/2021/1405271 (PMC8443362; doi:10.1155/2021/1405271)
Supplement: Supplementary Materials — Supplemental file 1 shows flow diagram of this randomized controlled trial. [file 1405271.f1.doc]

**
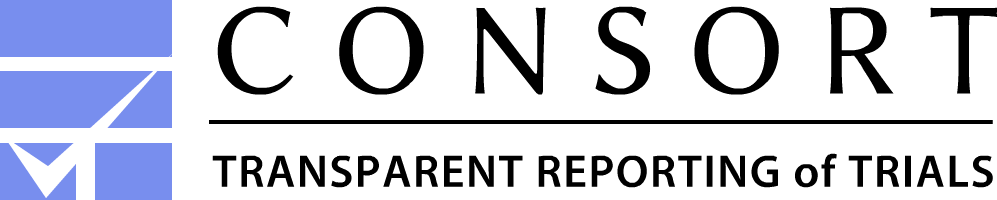
**

**CONSORT 2010 Flow Diagram**

**Allocation**

**Analysis**

**Follow-Up**

**Enrollment**

Assessed for eligibility (n= 196 )

Excluded (n= 16 )

♦  Not meeting inclusion criteria (n=10 )

♦  Declined to participate (n= 3 )

♦  Other reasons (n= 3 )

Analysed (n=54 )
♦ Excluded from analysis (give reasons) (n=6 )

Lost to follow-up (give reasons) (n=0 )

Discontinued intervention (give reasons) (n=0)

Allocated to intervention (n= 60 )

♦ Received allocated intervention (n=60 )

♦ Did not receive allocated intervention (give reasons) (n=0 )

Lost to follow-up (give reasons) (n= 0 )

Discontinued intervention (give reasons) (n=0 )

Allocated to intervention (n=60 )

♦ Received allocated intervention (n= 60 )

♦ Did not receive allocated intervention (give reasons) (n= 0 )

Analysed (n= 24 )
♦ Excluded from analysis (give reasons) (n=36)

Randomized (n=180 )
